# Ajmaline blocks *I*_Na_ and *I*_Kr_ without eliciting differences between Brugada syndrome patient and control human pluripotent stem cell-derived cardiac clusters

**DOI:** 10.1016/j.scr.2017.11.003

**Published:** 2017-12

**Authors:** Duncan C. Miller, Stephen C. Harmer, Ariel Poliandri, Muriel Nobles, Elizabeth C. Edwards, James S. Ware, Tyson V. Sharp, Tristan R. McKay, Leo Dunkel, Pier D. Lambiase, Andrew Tinker

**Affiliations:** a William Harvey Research Institute, Barts and the London School of Medicine and Dentistry, Queen Mary University of London, London, UK; b Barts Cancer Institute, Barts and the London School of Medicine and Dentistry, Queen Mary University of London, London, UK; c National Heart and Lung Institute, NIHR Royal Brompton Cardiovascular BRU, Imperial College London, London, UK; d School of Healthcare Science, Manchester Metropolitan University, Manchester, UK; e Institute of Cardiovascular Science, UCL and Barts Heart Centre, London, UK

**Keywords:** Brugada syndrome, hiPSC-cardiomyocytes, Ajmaline, *I*_Na_, *I*_Kr_, Activation-recovery interval, AP, Action Potential, APD, Action Potential Duration, Br1, Brugada Type 1 ECG, BrS, Brugada Syndrome, cFPD, Corrected Field Potential Duration (Bazett's formula), CM, Cardiomyocyte, ECG, Electrocardiogram, FP, Field Potential, FPD, Field Potential Duration, hPSC, Human Pluripotent Stem Cell, hiPSC, Human Induced Pluripotent Stem Cell, HDFs, Human Dermal Fibroblasts, MEA, Multi Electrode Array, NGS, Next Generation Sequencing, RMP, Resting Membrane Potential, VF, Ventricular Fibrillation

## Abstract

The class Ia anti-arrhythmic drug ajmaline is used clinically to unmask latent type I ECG in Brugada syndrome (BrS) patients, although its mode of action is poorly characterised.

Our aims were to identify ajmaline's mode of action in human induced pluripotent stem cell (hiPSC)-derived cardiomyocytes (CMs), and establish a simple BrS hiPSC platform to test whether differences in ajmaline response could be determined between BrS patients and controls.

Control hiPSCs were differentiated into spontaneously contracting cardiac clusters. It was found using multi electrode array (MEA) that ajmaline treatment significantly lengthened cluster activation-recovery interval. Patch clamping of single CMs isolated from clusters revealed that ajmaline can block both *I*_Na_ and *I*_Kr_.

Following generation of hiPSC lines from BrS patients (absent of pathogenic *SCN5A* sodium channel mutations), analysis of hiPSC-CMs from patients and controls revealed that differentiation and action potential parameters were similar. Comparison of cardiac clusters by MEA showed that ajmaline lengthened activation-recovery interval consistently across all lines.

We conclude that ajmaline can block both depolarisation and repolarisation of hiPSC-CMs at the cellular level, but that a more refined integrated tissue model may be necessary to elicit differences in its effect between BrS patients and controls.

## Highlights

• hiPSC lines generated and differentiated from BrS patients lacking SCN5A mutations
• Ajmaline lengthens the activation-recovery interval of hPSC cardiac clusters
• Ajmaline effect consistent between BrS patient and control hPSC cardiac clusters
• Patch clamp analysis of hiPSC-CMs reveals ajmaline blocks both *I*_Na_ and *I*_Kr_

## 1 Introduction

Brugada syndrome (BrS) is a cardiac arrhythmic syndrome and can cause ventricular fibrillation (VF) and sudden cardiac death. BrS is predominantly characterised by right bundle branch block, elevated J-point and coved ST segment of an electrocardiogram (ECG), with fibrillation and premature ventricular contractions often originating from the right ventricular outflow tract (RVOT) (Brugada and Brugada, 1992, Morita et al., 2003). Two specific hypotheses have been proposed to account for the Brugada ECG pattern - the depolarisation hypothesis, highlighting the importance of right ventricular activation delays, and the repolarisation hypothesis, focusing on transmural differences in action potential duration (Meregalli et al., 2005).

The class IA anti-arrhythmic drug ajmaline is used as a diagnostic pharmacological challenge in suspected cases of BrS (Rolf et al., 2003), however the mechanism of action is not fully established, and there has long been debate about the precision of its diagnostic effect (Brugada et al., 2003). Some studies in non-human or non-cardiac cell lines have indicated that ajmaline inhibits various currents, including *I*_Na_, *I*_to_ or *I*_Kr_ (Bébarová et al., 2005, Kiesecker et al., 2004). Furthermore, a study recently found that ajmaline elicited a Brugada type I ECG pattern in 27% of atrioventricular nodal re-entrant tachycardia patients and even in 5% of control individuals (Hasdemir et al., 2015). The exact pathophysiological mechanism whereby ajmaline provokes the BrS ECG phenotype is thus unclear.

It is clear that in some cases BrS can be hereditary, and in these cases occurs predominantly due to loss-of-function mutations in the cardiac sodium channel *SCN5A* (Mizusawa and Wilde, 2012). Mutations have also been identified in sodium channel beta subunits and in other ion channels such as the L-type Ca^2 +^ channel, however these are rare (Mizusawa and Wilde, 2012). There is a diversity of other associated genes with rare variants of sometimes disputed contribution (Le Scouarnec et al., 2015), as well as combinations of common variants likely to be contributing as disease modifiers (Bezzina et al., 2013). In total, a monogenic aetiology can be reasonably supported in only ~ 25% of patients (Wilde and Behr, 2013). Thus in the majority of patients there is scant data supporting a strong genetic predisposition. Furthermore, in addition to electrophysiological effects at the single cell level there may be structural changes that are critical for disease pathogenesis (Catalano et al., 2009, Papavassiliu et al., 2010).

The developments in human somatic cell reprogramming to induced pluripotent stem cells (hiPSCs) (Okita et al., 2011, Takahashi et al., 2007) and targeted differentiation of human pluripotent stem cells (hPSCs) to cardiomyocytes (CMs) (Burridge et al., 2011, Minami et al., 2012) have hugely empowered in vitro modelling of hereditary cardiac disease. Progress has been made in establishing hiPSC models for certain arrhythmias such as Long QT syndrome (Matsa et al., 2011, Sala et al., 2016), however diseases such as BrS present a more variable phenotype and polygenic background, with the potential added complexity of *endo*-epicardial tissue heterogeneities.

Here we aimed to address some of these issues by generating a simple cardiac model for the action of ajmaline in a BrS context, comprised of differentiation and electrophysiological analysis of hPSC-cardiac clusters/CMs from BrS patients and controls. Specifically we focus on patients without mutations in the cardiac sodium channel *SCN5A*, reflecting the predominant clinical population, and how they respond to ajmaline challenge.

## 2 Materials and methods

### 2.1 Consent and ethics

Work with human embryonic stem cells (hESCs) was reviewed and approved by the UK's Steering Committee For The Stem Cell Bank And For The Use Of Stem Cell Lines (reference number SCSC13–25). Use of patient samples following informed consent was approved by the UK's National Research Ethics Service (13/LO/0224).

### 2.2 Brugada subject identification

BrS subjects were identified from specialist inherited arrhythmia clinics, recruiting individuals with a history of BrS and out-of-hospital cardiac arrest or family members of sudden arrhythmic death syndrome (SADS) victims, who met the diagnostic criteria according to the 2013 inherited arrhythmia consensus document with either a spontaneous resting type 1 Brugada ECG pattern or positive ajmaline challenge test on screening (Priori et al., 2014).

### 2.3 Next generation sequencing (NGS) of patient and control genomic DNA

Following phenol:chloroform extraction of genomic DNA from blood samples, DNA libraries were prepared using the Illumina TruSight Cardio system (Pua et al., 2016) according to manufacturer's laboratory protocols, and sequenced on the Illumina NextSeq. Twelve genes in which variants had been previously reported as causative of BrS were analysed, together with a further 26 genes reportedly linked to other inherited arrhythmia syndromes (Table 2). Rare protein-altering variants were identified as those occurring at a frequency of ≤ 0.0001 (1 in 10,000) both in the ExAC database and in a cohort of healthy volunteers sequenced on the same platform (Lek et al., 2016). For further details please see Supplementary methods.

### 2.4 Reprogramming and maintenance of hPSCs

Human dermal fibroblast (HDF) cultures were derived from 4 mm skin punch biopsies from BrS patient and control individuals following overnight digestion with collagenase I (Sigma, USA). After two to five passages, HDFs were reprogrammed by lentiviral transduction of a polycistronic vector hSTEMCCA (Somers et al., 2010) or nucleofection of three plasmids containing reprogramming factors (Okita et al., 2011). Colonies were isolated and expanded clonally in DMEM:F12 medium containing 20% knock-out serum replacement (Gibco, UK) and 20 ng/ml FGF2 (Peprotech, USA) on mitotically inactivated mouse embryonic fibroblasts (MEFs), and characterised for pluripotency. hPSC lines were generally maintained and expanded on MEFs, and transitioned onto Matrigel in mTeSR for several passages prior to differentiation.

### 2.5 Cardiac differentiation of hPSCs

We adapted the protocol published by Burridge and colleagues (Burridge et al., 2011). hPSCs were enzymatically dissociated with Accutase (Thermo Fisher, USA) and plated at a density of 1.5–2 × 10^6^/75 cm^2^ flask pre-coated with Matrigel (Corning, USA) in mTeSR™1 (Stem Cell Technologies, Canada) + 5 μM Y-27632 (Tocris, UK). The next day (differentiation day 0–1, “D0”), cells were dissociated and resuspended at a density of 7 × 10^4^ cells/ml in RPMI-Growth Factor (RGF) medium (for media formulation please see Supplementary table S2). Cells were pipetted into conical bottom 96-well plates (100 μl per well), and centrifuged at 950*g* for 5 mins to aggregate the cells into clusters. On D2, medium was exchanged with 100 μl RPMI-Serum (RS) medium, and on D3 this was again replaced with fresh RS medium but containing SB431542 (Tocris, UK). On D4, medium was changed for 150 μl RPMI-ITS (RI) medium containing inhibitors KY02111 (Tocris) and XAV939 (Sigma, UK) (Minami et al., 2012) and clusters were transferred to round bottom 96-well plates. From D6 and henceforth RI medium without inhibitors was used to maintain the clusters, with medium changed every three days. In some experiments, clusters were transferred and pooled into 6-well plates from D14. For metabolic enrichment of CMs (Tohyama et al., 2013), cardiac clusters were maintained in RI comprised of RPMI without glucose (Gibco) and 4 mM sodium lactate (Sigma) between D14–21.

### 2.6 Multi electrode array (MEA) analysis of hPSC-cardiac clusters

At weeks 6–8 of differentiation, two or three spontaneously contracting clusters per analysis were loaded onto gelatin or Matrigel-coated 60PedotMEA200/30iR-Au MEAs (containing 60 gold coated electrodes, 200 μm spaced) (Multi Channel Systems, Germany) in RI medium containing 20% FCS (to promote attachment). After two days, clusters were analysed using the MEA2100 system with an HS60 headstage, and signals were recorded using MC_Rack software (all from Multi Channel Systems). Sampling frequency was 10,000 Hz, and channels with a detectable signal in the range of ± 50 μV were selected and recorded. Basal medium of RPMI containing pen/strep and 5% FCS +/− drug dose was superfused across clusters at a rate of ~ 1.5 ml/min, with the headstage and superfused medium maintained at 37 °C throughout. One minute baseline recordings were taken after at least 15 mins superfusion of basal medium, and all subsequent 1 min drug dose recordings were taken following 6 mins superfusion. Drugs tested had stock solutions as follows: ajmaline (Carinopharm GmBH, Germany) at 15.32 mM in phosphate saline solution (ultrapure for infusion), mexiletine (Sigma) at 100 mM in methanol, dofetilide (Santa Cruz Biotechnology, USA) at 100 mM in DMSO, isoprenaline (Sigma) at 10 mM in dH_2_O.

Raw electrode traces were converted using MC_DataTool (Multi Channel Systems) and analysed using Clampfit 10.6 (Molecular Devices, USA). To compare field potentials (FPs), ensemble average signals were generated from 1 min recordings using CardioMDA software, available from Pradhapan et al. (Pradhapan et al., 2013). Signals with a baseline beat rate of at least 25 beats/min (bpm) were averaged using a 90% correlation factor. Activation-recovery interval was taken as the field potential duration (FPD), and was determined for each ensemble trace manually based on the interval between the activation and the recovery peaks, as used previously (Egashira et al., 2012, Harris et al., 2013, Matsa et al., 2011). FPD values were then corrected (cFPD) using Bazett's formula (BAZETT, 1997, Sagie et al., 1992). The maximum negative gradient of the FP activation complex downslope (minimum velocity, dV/dt_min_), used as an indication of depolarisation (Qu and Vargas, 2015), was determined from ensemble average traces using Matlab. Statistical analyses were performed using SPSS 23 (IBM, USA) before or after baseline normalisation of individual electrodes (as indicated).

### 2.7 Patch-clamp analysis of hiPSC-CMs

For dissociation of cardiac clusters to single cells, please see Supplementary methods.

Patch clamp recordings were performed with an Axopatch 200B amplifier (Axon Instruments, USA) at either at room temperature 22 ± 2 °C (current-clamp and *I*_Na_) or at 36.5 ± 0.5 °C (*I*_Kr_). Patch-clamp recordings were performed using fire-polished pipettes with a resistance of 2–4 MΩ pulled from filamented borosilicated glass capillaries (Harvard Apparatus, 1.5 mm OD × 1.17 mm ID). Pipette capacitance was reduced by coating the tip with SigmaCote (Sigma). Data were acquired and analysed by using a Digidata 1440A interface (Axon Instruments) and pCLAMP software. For sodium currents (*I*_Na_) all recordings were done in a low sodium extracellular solution containing (mM): NaCl 25, CsCl 120, CaCl_2_ 1, MgCl_2_ 1, HEPES 5 and Glucose 10 (buffered to pH 7.4 with CsOH). The intracellular solution was (mM): NaCl 5, CsCl 125, MgATP 2, EGTA 10 and HEPES 20 (buffered to pH 7.2 with CsOH). To characterize the voltage dependency of the peak *I*_Na_, cells were held at − 90 mV, and 200 ms steps were applied from − 90 mV to + 30 mV in 5 mV increments. The interval between the voltage steps was 3 s.

For *I*_Kr_ and current clamp analysis of action potential (AP), pipette (intracellular) solution contained (mM): 110 K-d-gluconate, 20 KCl, 10 NaCl, 2 EGTA, 10 HEPES, 1 MgCl_2_, 0.3 GTP, and 2 MgATP (pH 7.4 with KOH). Extracellular solution (Tyrode's) contained (mM): 135 NaCl, 5.4 KCl, 5 HEPES, 1 MgCl_2_, 0.33 NaH_2_PO_4_, 2 CaCl_2_ and 10 Glucose (pH 7.4 with NaOH). For *I*_Kr_ analysis, the baseline ‘*I*_K_’ current was recorded in the presence of 5 μM nifedipine and 3 μM HMR1556 to block *I*_CaL_ and *I*_Ks_ respectively. Two minutes after drug addition, *I*_Kr_ was elicited by stepped depolarisations from − 40 to + 30 mV for 2 s in 10 mV increments, followed by a repolarising pulse back to − 40 mV for 2 s (to measure *I*_Kr_ tail currents). Ajmaline (100 μM) (with nifedipine 5 μM and HMR1556 3 μM) was then perfused for 2 min and a second I/V protocol was run. After the second I/V protocol ajmaline perfusion was stopped and the recovery of the *I*_Kr_ current monitored by successive I/V protocols. If *I*_Kr_ tail current returned this was then blocked by the addition of dofetilide 1 μM (an *I*_Kr_ specific blocker) (with nifedipine 5 μM and HMR1556 3 μM) for 2 min and a final I/V protocol recorded. Series resistance was compensated by ~ 70% using the amplifier circuitry. Current-voltage relationships were determined by normalising the maximal current densities at the end of each pulse potential to cell capacitance (pA/pF). Peak tail current density (PTCD) was determined by normalising the maximal current densities of the peak tail currents in response to each pulse potential to cell capacitance (pA/pF). For current-clamp analysis, once the whole-cell configuration had been achieved, APs were recorded in the current-clamp mode. For cells that were spontaneously firing we first recorded their activity and then applied a trigger (500pA-1.8 nA depending on cell size and response) at a frequency of 1 Hz. For cells that were quiescent we applied a trigger (500pA-1.8 nA depending on cell size and response) at a frequency of 1 Hz. Cells with a resting membrane potential (RMP) of above − 30 mV were not included in the analysis because they were considered to be either too immature or non-cardiac. APs were Liquid Junction Potential (LJP) corrected (LJPc). The LJPc (13.4 mV) was calculated using the Clampex Junction Potential Calculator. Experiments were analysed using Clampfit, and Origin, and data statistically compared using SPSS 23.

AP morphologies were categorised based on the ratio of AP duration (APD)_90/50_, as described and used previously (Matsa et al., 2011). APs with a plateau phase as distinguished by a ratio ≤ 1.4 were considered ventricular, those with ≥ 1.7 atrial, and 1.4 to 1.7 nodal-like.

### 2.8 Reverse transcription PCR (RT-PCR)

The relative expression of targets was determined by qRT-PCR using the 2^− ΔCt^ method (Schmittgen and Livak, 2008), normalised to *GAPDH* as the housekeeping gene. For further details please see Supplementary methods.

### 2.9 Immunofluorescence microscopy and flow cytometry

For details of protocols and reagents please see Supplementary methods.

## 3 Results

### 3.1 Generating and differentiating hiPSC cardiac clusters

**Fig. 1:**
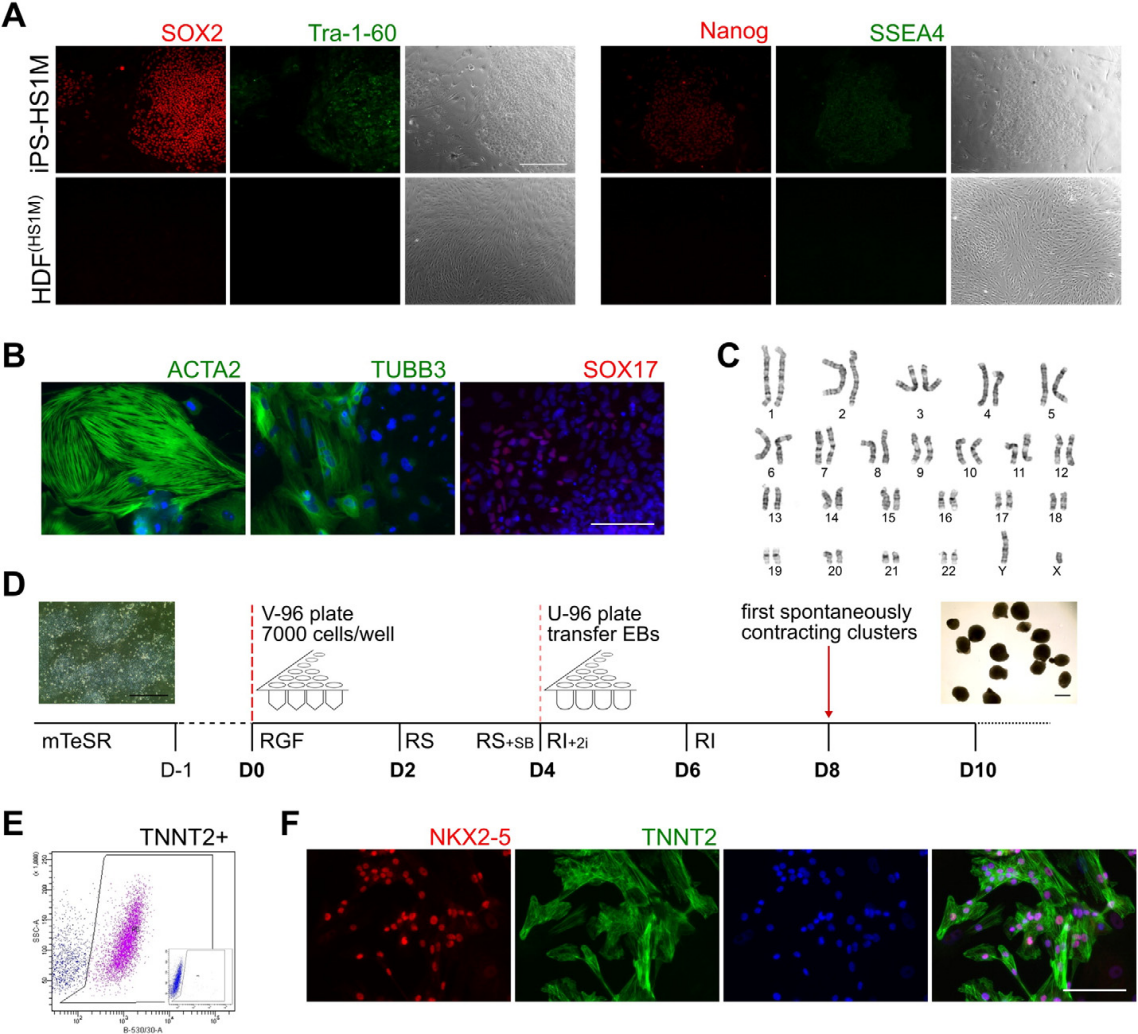
Reprogramming of hiPSCs and differentiation to cardiac clusters. A) Immunocytochemistry of pluripotency associated factors in iPS-HS1M hiPSCs following reprogramming from HDFs. Bar = 100 μm. B) Immunocytochemistry of lineage markers of mesoderm (ACTA2), neurectoderm (TUBB3) and endoderm (SOX17) plus DAPI staining (blue) following non-directed EB differentiation and outgrowth. Bar = 100 μm. C) G banding of iPS-HS1M, indicating a normal karyotype following reprogramming. D) Schematic with phase contrast images inset of hiPSC differentiation to cardiac clusters. For media formulations see Supplementary table S2. Bar = 500 μm. E) Flow cytometry analysis of TNNT2 expression in iPS-HS1M at differentiation day 21, with IgG isotype control inset. F) Immunocytochemistry of NKX2-5 and TNNT2 plus DAPI at day 28 of differentiation following partial dissociation of iPS-HS1M cardiac clusters. Bar = 100 μm.

In order to establish a simple in vitro model to investigate the effects of ajmaline, we began by generating a control hiPSC line from a consenting adult male without a BrS phenotype, designated HS1M. HDFs were reprogrammed using a polycistronic lentiviral vector containing Yamanaka factors (Somers et al., 2010), with several colonies individually isolated in the first passage and clonally expanded. Tra-1-60 live cell staining was used to screen colonies at passage 2 (data not shown). Positive clonal lines were expanded and screened for consistent cellular morphology, expression of pluripotency-associated markers, normal karyotype, and embryoid body (EB) differentiation to all three germ layers (Fig. 1A–C). One line was selected for differentiation experiments, designated iPS-HS1M.

To generate cardiac material readily usable for electrophysiological analysis, we adapted features of the EB/cardiac cluster protocol published by Burridge and colleagues (Burridge et al., 2011), additionally employing the Activin/Nodal receptor inhibitor SB431542 on day 3, followed by the two canonical WNT inhibitors KY02111 and XAV939 from the report by Minami (Minami et al., 2012) on days 4–6 (Fig. 1D). Cluster morphology was consistent, with beating observed from day eight onwards (Movie 1). Flow cytometry analysis following dissociation of clusters at day 21 showed a cardiac Troponin T (TNNT2) expressing population of 52.2% ± 12.2 (n = 4) (Fig. 1E). CMs were observed by immunofluorescence microscopy, with sarcomeric structures evident by TNNT2 staining, as well as nuclear co-staining of the pan-cardiac marker NKX2-5 (Fig. 1F).

### 3.2 Ajmaline reduces activation and lengthens activation-recovery interval of hiPSC cardiac clusters

**Fig. 2:**
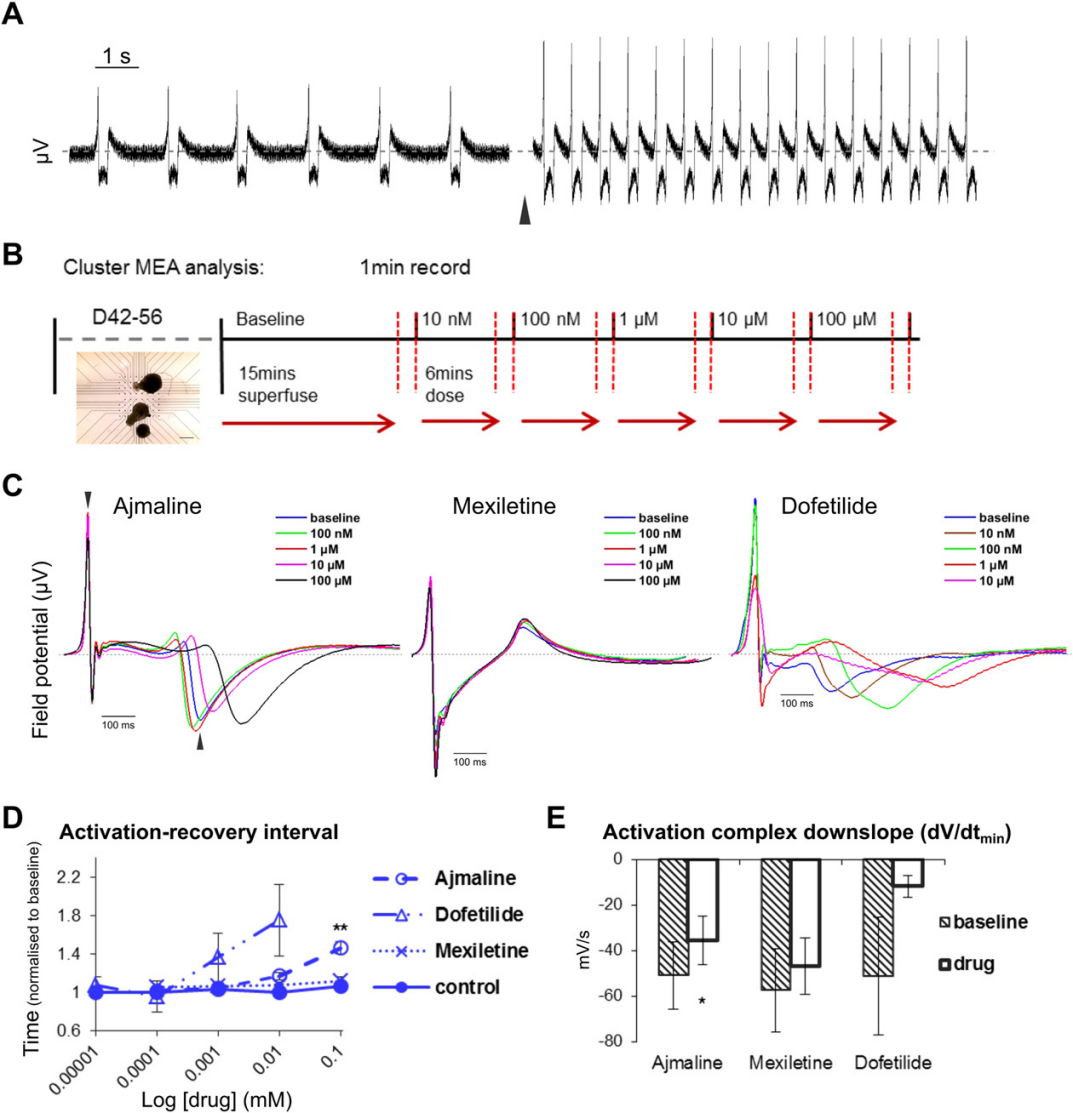
Effects of ion channel inhibitors on field potential (FP) of hiPSC cardiac clusters. A) Raw FP signal from a single MEA electrode, showing the effect following 6 mins superfusion of 500 nM isoprenaline (black arrow). B) Schematic of MEA protocol, with phase contrast image inset of cardiac clusters loaded onto an MEA. Bar = 500 μm. C) Representative ensemble FP signals at individual electrodes, showing effect of increasing doses of ajmaline, mexiletine or dofetilide on iPS-HS1M clusters. Arrow heads indicate examples for time points of activation and recovery peaks, to calculate cFPD. Grey dashed line shows 0 μV. D–E) Mean iPS-HS1M cluster activation-recovery interval over increasing drug doses (D), or activation complex downslope gradient dV/dt(minimum) at highest drug dose (E), showing effect of ajmaline (n ≥ 9), mexiletine (n ≥ 9) and dofetilide (n ≥ 4, some clusters ceased beating or were too inconsistent to generate ensemble averages at higher doses) normalised to baseline, error bars show ± s.e.m. One way ANOVA followed by Tukey HSD (D) or paired *t-*test (E), ** = p ≤ 0.01 or * = p ≤ 0.05 respectively vs baseline before normalisation.

In order to validate the hiPSC cardiac clusters and determine the effects of ion channel inhibitors, we loaded iPS-HS1M cardiac clusters onto MEAs after six to eight weeks of differentiation, to measure the FP of spontaneous contraction. Cardiac clusters were responsive to β-adrenergic stimulation, showing a greatly increased beat rate following 500 nM isoprenaline superfusion (Fig. 2A). Drugs were administered via superfused medium in increasing doses for 6 mins each followed by 1 min recordings (Fig. 2B), sufficient that each dose had a stable effect on clusters (Supplementary Fig. S1A—B). To generate representative data sets, recordings of drug dose effects vs baseline were taken from multiple clusters across at least eight electrodes, from three or more separate MEA analyses and where possible from several independent differentiation experiments.

Superfusion of basal medium alone over successive 6 min intervals had no effect on cFPD, however treatment with increasing doses of ajmaline consistently lengthened cFPD, significantly increasing the activation-recovery interval by 1.46 fold at 100 μM (Fig. 2C–D). We also enriched our clusters for CM content using sodium lactate (Tohyama et al., 2013) and observed near identical effects of ajmaline on the activation-recovery interval (Supplementary Fig. S1C–D). In comparison with ajmaline, the Na_v_1.5 channel inhibitor mexiletine caused no observable effect on cFPD (Fig. 2C–D) until 1 mM, at which point complete cessation of contraction occurred (data not shown). We also tested dofetilide, the potent rapid delayed rectifying potassium current (*I*_Kr_) blocker, and found that it had a dramatic effect on spontaneous contraction of clusters, causing the cFPD to lengthen at doses several orders of magnitude lower than ajmaline (Fig. 2C–D). Dofetilide also appeared to cause an increased beat rate in clusters, whereas again mexiletine had no observable effect (until cessation at 1 mM) and ajmaline tended to slow cluster beating at 100 μM (Supplementary Fig. S1E). To determine what effects these drugs had on cluster activation, we compared the downslope gradient of the FP activation complexes at baseline and following the highest drug dose. Ajmaline slightly but significantly reduced the gradient of activation, whereas mexiletine had no clear effect, and dofetilide appeared to greatly reduce the activation complexes (although also causing inconsistent beating or cessation at the highest dose) (Fig. 1C, E). These data suggest that ajmaline may affect depolarisation as well as cause lengthening of cFPD similarly to dofetilide via inhibition of repolarising currents such as *I*_Kr_ in spontaneously contracting hiPSC cardiac clusters.

### 3.3 Ajmaline blocks *I*_Na_ and *I*_Kr_ in hiPSC-CMs

Since ajmaline at higher doses had such a clear and consistent effect on hiPSC cardiac cluster activation-recovery interval, we sought to identify whether ajmaline is able to inhibit *I*_Kr_ in hiPSC-CMs, as well as confirm it can inhibit *I*_Na_ on which its clinical diagnostic use is based (Sarquella-Brugada et al., 2015). Ajmaline has been detected in subjects showing idioventricular rhythm at plasma concentrations of 9.1–46.17 μM (Padrini et al., 1993), so we chose the higher dose from our MEA analysis of 100 μM to investigate isolated iPS-HS1M CMs by patch clamping.

**Fig. 3:**
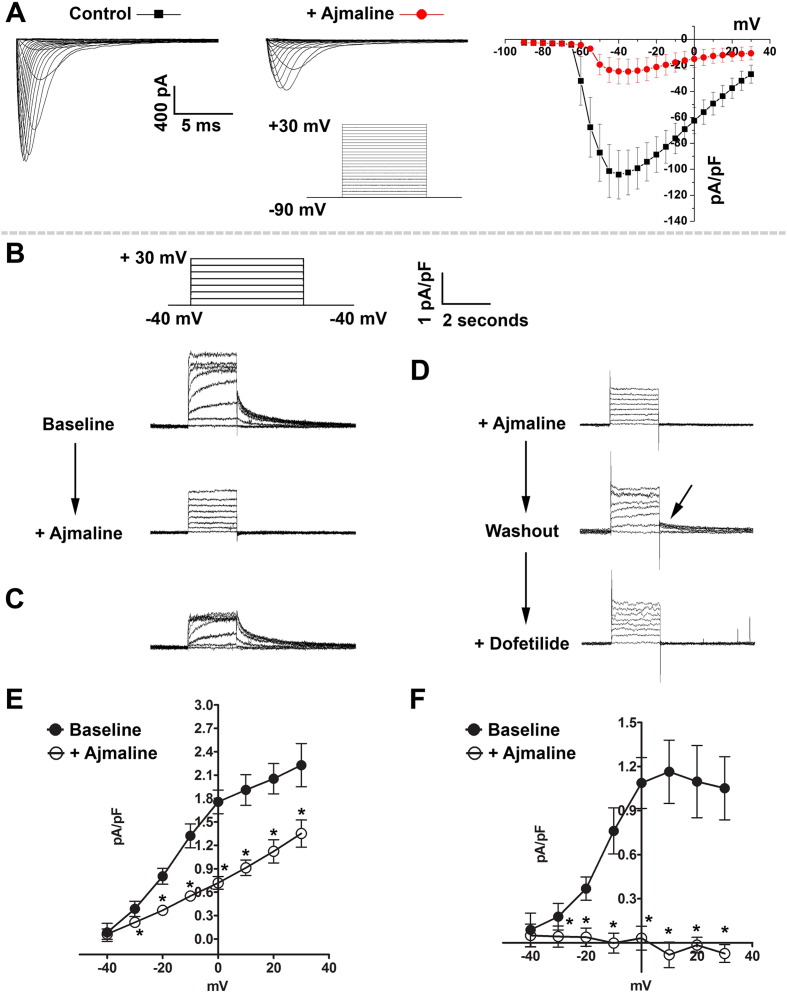
Ajmaline blocks both *I*_Na_ and *I*_Kr_ in hiPSC-CMs. A) Representative *I*_Na_ traces and current density (pA/pF) under control conditions and after application of ajmaline (100 μM), n = 15 cells. B) Representative traces showing the effect of application of ajmaline (100 μM) on *I*_Kr_ tail currents. The voltage protocol and scale are inset. C) Digital subtraction of the current blocked by ajmaline. D) In some cells the *I*_Kr_ tail current partially recovered after removal (washout) of ajmaline (indicated by arrow) and this could then be blocked by the addition of the *I*_Kr_ specific blocker dofetilide (1 mM). E) Current density before and after ajmaline application. F) Peak-tail current density (PTCD) before and after ajmaline application. * = p ≤ 0.05 (paired *t-*test) compared to baseline value before ajmaline addition, n = 8 cells. Data displayed as mean ± s.e.m.

Following administration of ajmaline, we observed marked inhibition of *I*_Na_ in hiPSC-CMs compared to baseline control (Fig. 3A, B), and this inhibition was partially recovered after washout: pA/pF control = − 110 ± 18.7, ajmaline = − 15.4 ± 4.4, washout = − 55 ± 11.6. When isolating outward potassium currents, the perfusion of ajmaline at 100 μM also resulted in an approximate 50% reduction in current density and a complete disappearance of peak tail current density compared to baseline control, which could be partially recovered after washout (Fig. 3C–F). The complete loss of tail current indicates that ajmaline acts to completely block *I*_Kr_ at 100 μM. These data therefore show that ajmaline inhibits both sodium and potassium currents in hiPSC-CMs, and support our observation that ajmaline lengthens the activation-recovery interval of hiPSC cardiac clusters by inhibiting *I*_Kr_.

### 3.4 BrS patient selection and genotyping

**Fig. 4:**
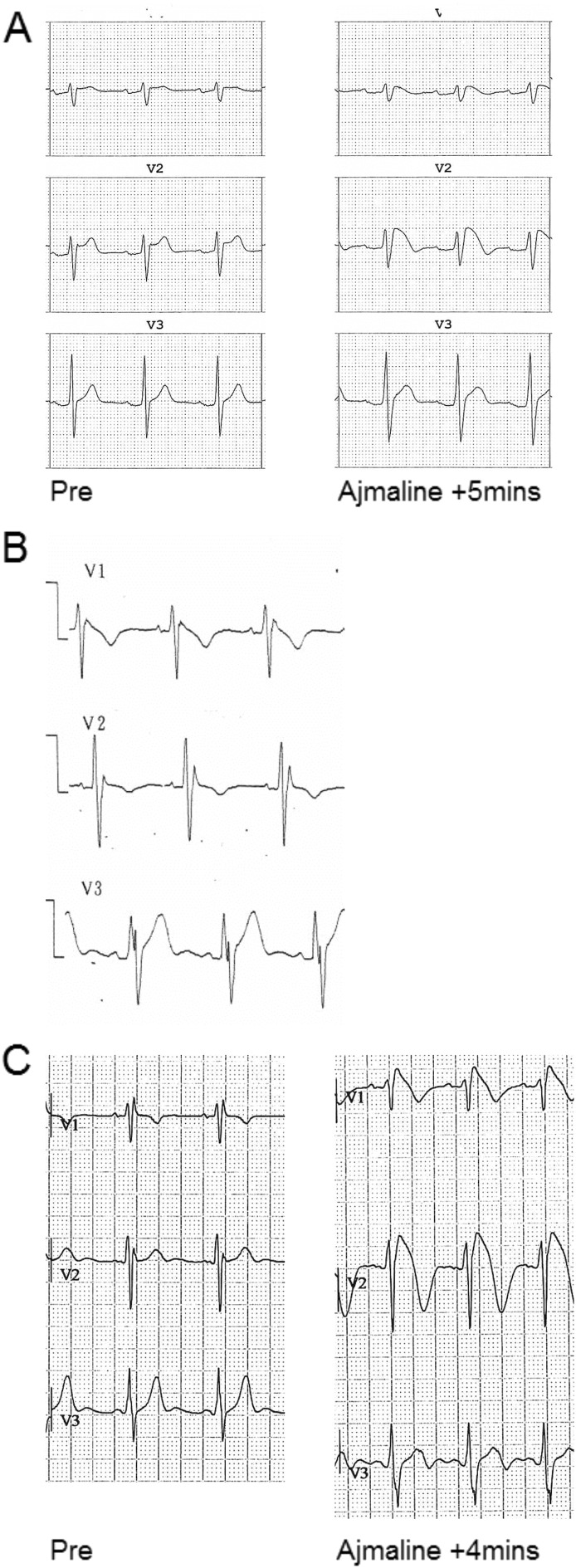
BrS patient ECGs. A–C) ECGs of patients BR1-P3M (A), BR1-P5M (B) and BR1-P6M (C), with an abnormal ST segment and T wave evident in precordial lead 1 (V1) and/or V2, either spontaneously (B), or after administering 1 mg/kg ajmaline (A, C).

Having established that MEA analysis could reveal the actions of ajmaline on hiPSC cardiac clusters, we sought to create a BrS hiPSC platform that would be representative of BrS genetic aetiology. Consenting patients were selected from a cohort affiliated with the former UCLH Heart Hospital, UK. Patients were screened based on a strong BrS phenotype, and absence of putative pathogenic *SCN5A* mutations. Fitting these criteria were three male patients (mean age 42.4 ± 12.9 years), designated BrS patient 3 male (BR1-P3M), BR1-P5M and BR1-P6M. Patients exhibited a BrS ECG pattern in line with HRS/EHRA/APHRS consensus guidelines (Priori et al., 2014) in ≥ 1 precordial lead either spontaneously or following ajmaline treatment, and in some cases had suffered VF events (Fig. 4A–C, Table 1). NGS of BrS patients and our control HS1M using the Illumina TruSight Cardio panel (Pua et al., 2016) did not reveal any rare protein altering variants in BrS genes as defined by ≤ 0.0001 frequency in the ExAC database (see Methods). We note a variant in *PKP2* (ExAC 0.00012) in BR1-P3M that has previously been linked to BrS, but has also been repeatedly observed at low frequency in the population, and is considered of unknown significance or likely benign. Otherwise, NGS data indicated that our individuals were absent of rare protein-altering and likely pathogenic variants in *SCN5A*, other BrS associated genes, or inherited arrhythmia-related targets (see Table 2).

**Table 1:** Subjects used in study.

Individual designation	Disease state	Age	Clinical notes	Arrhythmia related rare variants detected by NGS
HS1M	–	39	–	–
BR1-P3M	Br1	68	Ajm inducible elevation + coved S-T leading to VF, occasional spont.	***PKP2*** – nsSNP, c.302G > A, p.R101H, VUS (ExAC 0.00012)
BR1-P5M	Br1	28	VF arrest 16yo, spont. J-point elevation, not-quite-coved S-T, father similar ECG	–
BR1-P6M	Br1	30	Ajm inducible type I, occasional spont.	–

Abbreviations: Br1 – Brugada syndrome type I ECG, Ajm – ajmaline, spont. – spontaneous, VF – ventricular fibrillation, nsSNP – non-synonymous single nucleotide polymorphism, c. – DNA coding sequence, p.- protein sequence, VUS – variant of unknown significance.

**Table 2:** Gene list of general arrhythmia related targets within the TruSight Cardio panel (Pua et al., 2016) assessed in BrS patient and control individuals by NGS.

Reported BrS genes	SCN5A, CACNA1C, CACNA2D1, CACNB2, GPD1L, HCN4, KCND3, KCNE3, KCNJ8, RANGRF, SCN1B, SCN3B
Other inherited arrhythmia-related genes	KCNH2, KCNQ1, RYR2, ABCC9, AKAP9, ANK2, CALM1, CASQ2, CAV3, DSC2, EMD, GJA5, JPH2, KCNA5, KCNE1, KCNE2, KCNJ2, KCNJ5, LMNA, MYH6, NKX2-5, NPPA, PKP2, SCN4B, SNTA1, TRDN

### 3.5 BrS patient and control hPSC cardiac differentiation and AP characterisation

HDFs from our three patients were reprogrammed to hiPSCs using either the lentiviral vector as before or by nucleofection of reprogramming factors (Okita et al., 2011). Colonies were selected, clonally expanded, and characterised as before (Supplementary Fig. S2), from which four lines were selected: iBR1-P5M-L1 and -L9, iBR1-P6M-L1, and iBR1-P3M-N2 (− L[number] or –N[number] denoting lentiviral or nucleofection clone number).

**Fig. 5:**
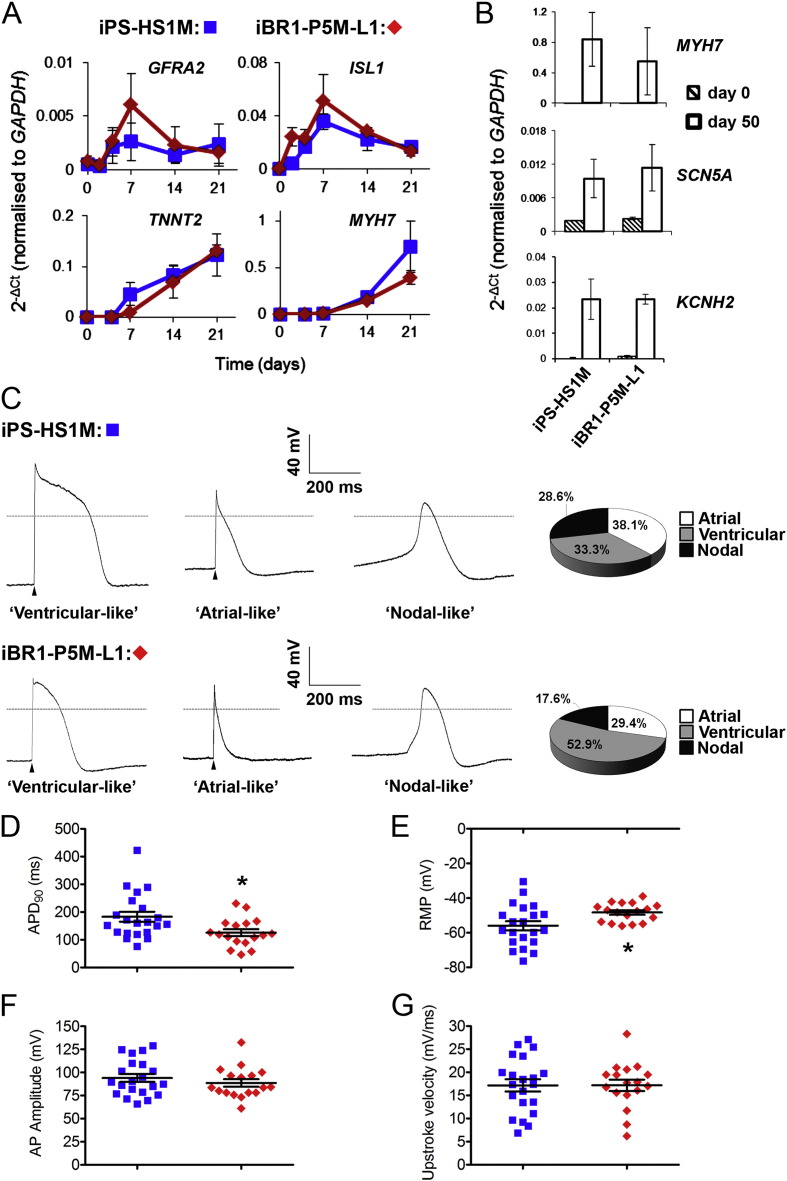
Comparison of BrS patient and control hiPSC-CM differentiation and APs. A–B) qRT-PCR analysis of marker expression during cardiac cluster differentiation of control iPS-HS1M and BrS patient iBR1-P5M-L1 lines. Data displayed from n = 3 (A) or n = 4 (B) independent differentiation experiments ± s.e.m. C–G) APs recorded from isolated iPS-HS1M (n = 21) and iBR1-P5M-L1 CMs (n = 17) at weeks 6–8 of differentiation. C) Representative traces and proportion of AP types (assigned as described in Methods). The arrow heads indicate that the AP was triggered (at 1 Hz). The grey dashed line indicates 0 mV. D–G) Collated AP data displaying the range of APD_90_ values (D), Resting Membrane Potential (RMP) (E), AP amplitude (F) and Upstroke Velocity (G). Data displayed as mean ± s.e.m. * = p ≤ 0.05 (independent *t-*test) vs control line iPS-HS1M.

To compare BrS patient and control hPSC lines we first used our directed differentiation protocol to generate cardiac clusters. The sequential use of small molecule inhibitors generated consistently high percentages of spontaneously contracting clusters across all lines, 88–95% at day 14, with a range of 37–71% range of TNNT2^+^ cells detected at day 21 (Supplementary Fig. S3B). To further validate cardiac differentiation, analysis of marker transcripts by qRT-PCR was performed on iPS-HS1M and iBR1-P5M-L1. Temporal induction of cardiac progenitor markers *GFRA2* and *ISL1* (Bu et al., 2009, Ishida et al., 2016) and CM markers *TNNT2* and *MYH7* was very similar over the first 21 days between the two cell lines (Fig. 5A). At day 50, clusters showed comparably high transcript profiles of *MYH7* and the sodium and potassium ion channel subunits *SCN5A* and *KCNH2* (Fig. 5B). These data do not reveal any gross difference in cardiac differentiation capacity between the BrS patient and control hPSCs using the cardiac cluster protocol.

To assess the composition of cardiac clusters and whether there may be any obvious intrinsic difference between BrS patient and control CMs, we performed current clamp analysis of single cells dissociated from clusters after six to eight weeks of differentiation, to determine their AP types and parameters. We decided to focus on only one patient and a control, given a recent publication by Veerman and colleagues finding almost no differences following comprehensive characterisation of membrane currents and APs in hiPSC-CMs from *SCN5A* mutation-negative BrS patients (Veerman et al., 2016). Patient BR1-P5M was chosen given his severe phenotype, having suffered spontaneous VF events leading to cardiac arrest during adolescence (Table 1). The AP morphologies, categorised based on APD_90/50_ ratios as used previously (Matsa et al., 2011) (see Materials and methods), suggested a similar range/composition of CM types from control and BrS clusters, with atrial or ventricular-like APs prevalent as well as a few APs with nodal/pacemaker-like morphology (Fig. 5C). The APDs were quite variable across CMs, with mean APD_90_ measurements of 183.0 ± 17.9 and 125.5 ± 12.4 ms for control and patient CMs respectively (Fig. 5D, n = 21 and 17 from two independent parallel differentiation experiments). RMP of iBR1-P5M-L1 CMs was slightly higher (Fig. 5E), however we saw no differences in AP amplitude (Fig. 5F) or AP upstroke velocity (Fig. 5G) between the two lines. These trends were also maintained when regarding only the ventricular-like CMs of the control and patient lines (n = 7 and 9). These data therefore indicated that although BrS patient and control hiPSC clusters were of mixed CM composition, they were consistent and comparable across most CM parameters.

### 3.6 Ajmaline treatment of BrS patient and control hPSC cardiac clusters

**Fig. 6:**
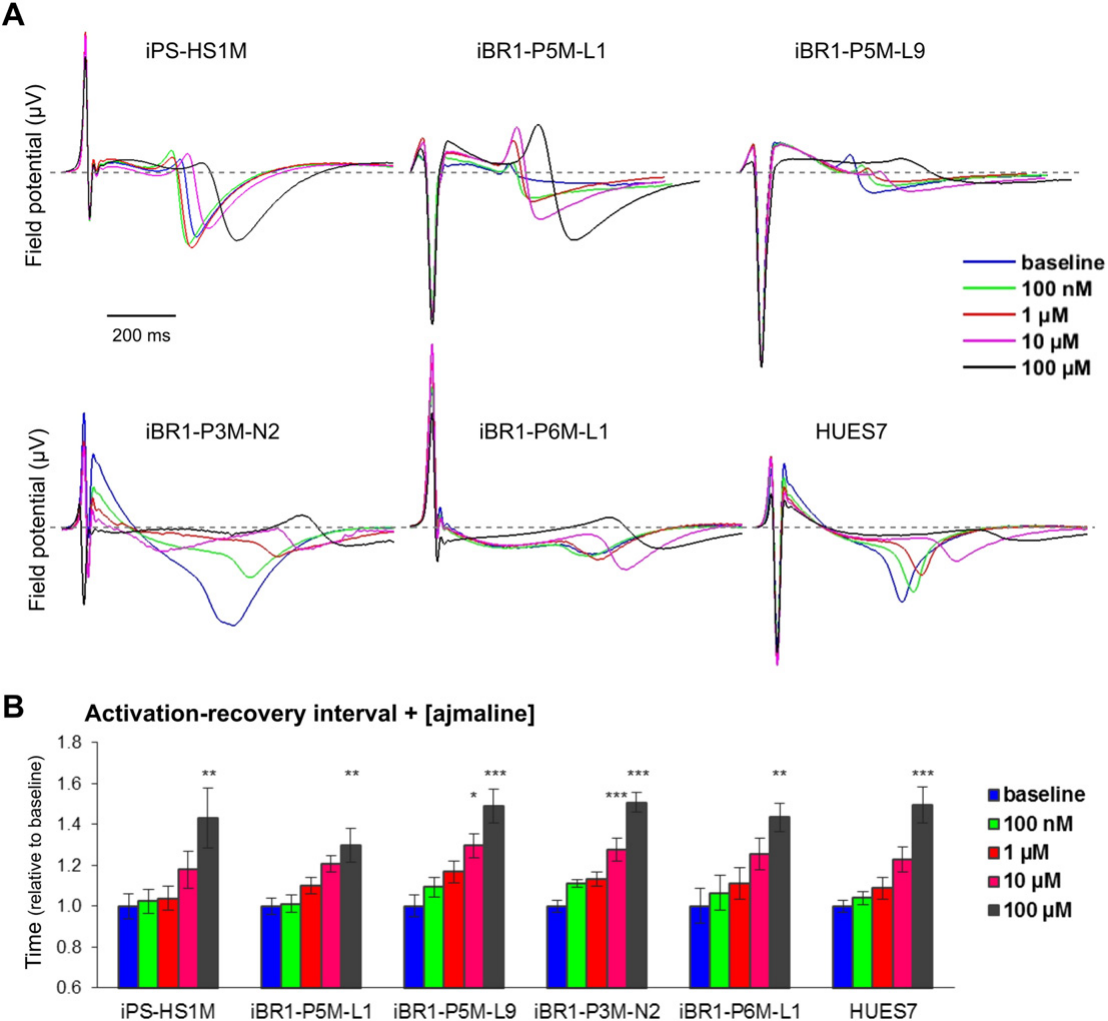
Effect of ajmaline on FP of BrS patient and control hPSC cardiac clusters. A) Representative ensemble FP signals at individual electrodes, showing effect of increasing doses of ajmaline on cardiac clusters from different hPSC lines. Grey dashed line shows 0 μV. B) Mean activation-recovery interval of clusters from different hPSC lines with increasing doses of ajmaline, calculated from cFPD relative to baseline reading. Data averaged from n ≥ 8 electrodes from n ≥ 3 MEA assays (see Supplementary table S1). One way ANOVA followed by Tukey HSD performed before normalisation, * = p ≤ 0.05, ** = p ≤ 0.01, *** = p ≤ 0.001 vs. baseline.

Since both we and others (Veerman et al., 2016) could identify few clear cell-intrinsic differences between hiPSC-CMs from controls and BrS patients absent of pathogenic *SCN5A* mutations, we decided to apply our multi-cellular cluster model to identify by MEA whether ajmaline could elicit a different response in activation-recovery interval between patients and controls. Cardiac clusters were loaded on MEAs and cFPD determined as before following increasing doses of ajmaline. Baseline cFPD across the different lines had a range of 281 to 395 ms, and although the patient lines P3M-N2 and P6M-L1 had significantly longer cFPD than HUES7 and/or P5M-L9, there was no significant difference found between the BrS patients or the control iPS-HS1M line (Supplementary table S1 and Supplementary Fig. S3C). Upon ajmaline addition, as observed with iPS-HS1M, all lines showed a steady increase in activation-recovery interval, with the cFPD becoming significantly longer at 100 μM and in some lines at 10 μM compared to baseline (Fig. 6). Complete cessation of spontaneous contraction occurred at 1 mM ajmaline (data not shown). Comparing the relative cFPD at 10 μM and 100 μM ajmaline across the patients and controls, we found no significant differences between any of the hPSC lines (one way ANOVA, p = 0.131 and 0.399 respectively). Similarly, upon comparison of the effect on cardiac cluster activation, no difference was found between the lines in the relative reduction of activation following the highest dose of ajmaline (one way ANOVA, p = 0.244, Supplementary Fig. S3D). Altogether, these data indicate that ajmaline consistently lengthens the activation-recovery interval as well as slightly reducing activation in hPSC cardiac clusters, however hPSC clusters from BrS patients absent of *SCN5A* arrhythmia-associated rare variants are neither more nor less sensitive to these effects compared to control.

## 4 Discussion

In this study we describe the action of ajmaline in a simple hiPSC model of human cardiac electrophysiology. Interestingly, in addition to inhibition of *I*_Na_, ajmaline significantly altered repolarisation through block of *I*_Kr_ in hiPSC-CMs, consistently lengthening the activation-recovery interval of cardiac clusters, emphasising that the action of ajmaline is broader than previously thought. Furthermore, we have generated hiPSCs from a number of patients with BrS. We selected patients with no genetic evidence of a sodium channel or other ion channel defect. We showed that there were few differences in electrophysiology between BrS patient and control hPSC-CMs or clusters, as assessed by AP morphology and MEA recordings in the absence or presence of ajmaline.

We challenged hiPSC-CMs with the drug ajmaline to elucidate its mode of action and parallel its diagnostic application in BrS. Plasma concentrations of 9.1–46.17 μM have been detected in subjects showing idioventricular rhythm (Padrini et al., 1993), hence our dose range was appropriate to study the drug's effects in the context of a disease model. We saw a prominent and consistent lengthening of cFPD in all cell lines including control and BrS patient cells. This did not occur with another class I anti-arrhythmic and sodium channel blocking drug, mexiletine, and more closely resembled the *I*_Kr_ blocker dofetilide. Furthermore, using patch clamping we isolated *I*_Na_ and *I*_Kr_ in hiPSC-CMs and showed that ajmaline blocked both currents. It is known that in addition to blocking *I*_Na_, ajmaline can block *I*_to_, *I*_CaL_ and *I*_Kr_, though this has been shown only in heterologous expressions systems or animal cells (Bébarová et al., 2005, Fischer et al., 2013, Kiesecker et al., 2004). However, this complex pharmacology is under appreciated and we demonstrate the ability of ajmaline to block *I*_Kr_ for the first time in a hiPSC-CM assay system. Interestingly, ajmaline and dofetilide both inhibited FP activation of cardiac clusters, and the latter also dramatically increased the beat rate, despite being a well characterised highly specific *I*_Kr_ blocker (Roukoz and Saliba, 2007). Both these effects may be caused indirectly due to the role *I*_Kr_ plays in repolarisation of hPSC-CMs in the context of a 3D cluster: hPSC-CMs are known to lack robust expression of *I*_K1_, hence *I*_Kr_ is required to set the resting membrane potential (Doss et al., 2012). When *I*_Kr_ is blocked a more depolarised diastolic potential results in a reduced current upon depolarisation, and CMs that are more readily triggered to depolarise are then also likely to beat faster (Qu and Vargas, 2015). Our data closely recapitulate the observations by Qu and Vargas regarding dofetilide, suggesting that FP recordings can be useful for determining drug effects on repolarisation, specifically *I*_Kr_, but may be limited in their ability to faithfully report direct effects on depolarisation in hPSC-CMs, particularly those lacking *I*_K1_.

Several recent studies have begun to address disease modelling of BrS using hPSCs. However, there has been a preferential focus on single cells and cases with SCN5A mutations. Liang and colleagues examined two patients, one with a missense mutation in a region important for voltage sensing and the other a base pair deletion leading to a premature stop (Liang et al., 2016). CMs showed reduced sodium current, reduced upstroke velocity of APs, triggered activity and abnormal Ca^2 +^ transients. The abnormal cellular behaviour was corrected by gene editing of the mutation. In a second recent study, Kosmidis and colleagues studied two nonsense mutations in SCN5A and also found reduced sodium currents and slower AP upstroke velocity (Kosmidis et al., 2016). Interestingly, readthrough therapy restored function to the channels in HEK293 cells but not hiPSC-CMs. In our study, we instead focussed on the common clinical scenario of SCN5A (and other ion channel) mutation-negative BrS patients to test whether electrophysiological differences could be revealed in this group. We began with AP measurements in one patient and one control as a broad assay for intrinsic cellular differences. Specifically, we saw no evidence for slowing of AP upstroke velocity, suggesting that the function of *I*_Na_ is not prominently affected. The slightly reduced APD of our BrS patient was similar to that observed in the report by Veerman et al. (Veerman et al., 2016). Our AP data agree with the findings of their detailed electrophysiological study of CMs from SCN5A mutation-negative BrS patients. This raises several key points, especially where models aim to elicit potentially modest differences at the single cell level, from a disease with complex aetiology and subtle or unclear pathophysiology. It is known that hPSC-CMs are more closely aligned in electrophysiological phenotype to foetal cells (Veerman et al., 2015). We cannot exclude the possibility that some differences may manifest themselves if CMs are matured to a more adult or right ventricular outflow tract specification.

We had aimed to determine whether the effect of ajmaline on whole cardiac clusters from BrS patients could provide an additional platform for investigation, especially given the drug's effects on our patients' ECGs and consistency in control hiPSC clusters. A multicellular system to model diseases such as BrS could provide much insight into arrhythmogenesis. Although we saw no differences with our simple cluster based system, a phenotype may be revealed in more precisely engineered cardiac microtissues, perhaps including co-culture with fibroblasts or endothelial cells, that more closely recapitulate the native tissue composition of the heart (Pellman et al., 2016), examples of which are rapidly developing (Giacomelli et al., 2017, Schaaf et al., 2016, Thavandiran et al., 2013, Tiburcy et al., 2017). Furthermore, this could be enhanced via selection for specific populations of CMs for example expressing a ventricular marker increasing the fidelity and potentially the sensitivity of such a model for diseases such as BrS.

The failure to find cellular electrophysiological abnormalities in this study and others supports the hypothesis that in a significant number of BrS patients there may not be a strong genetically inherited substrate arising from ion channel defects. There are two major hypotheses for the mechanism of disease which are not necessarily mutually exclusive. The characteristic ECG changes occur because there is either delayed depolarisation or early repolarisation in different regions of the right ventricular outflow tract (Antzelevitch, 2007, Coronel et al., 2005, Lambiase et al., 2009, Morita et al., 2008). These hypotheses are supported by the effects of fibrosis, tissue architectural changes or gap junction disruption to promote conduction delays, or cellular uncoupling effects in the myocardium which enable subtle differences in epi- and endocardial repolarisation behaviour to be dissociated and expressed on the surface ECG (Agullo-Pascual et al., 2014). The processes determining these could be genetic, environmental including age (BrS predominantly manifests in middle age) and the effects of previous myocarditis. It is being increasingly recognised that the epicardium of Brugada patients exhibits fibrotic changes and discontinuous conduction (Nademanee et al., 2015). This has led to an approach to ablate the tissue which would homogenise the substrate to abolish the ECG pattern and minimise the opportunity for local re-entry and wavebreak to develop (Brugada et al., 2015, Nademanee et al., 2011). Our data here support the contention that tissue architectural changes conspire to produce the Brugada phenotype independent of ion channel mutations. The use of ajmaline is proposed to magnify conduction delay, but our hiPSC data demonstrate that it is also possible that it may have differential effects on repolarisation.

## 5 Conclusions

In conclusion, we show using a simple hiPSC cardiac assay system that ajmaline exhibits previously undescribed effects on activation-recovery interval via *I*_Kr_ block, in addition to the inhibition of *I*_Na_. However, the system could not reveal electrophysiological differences between hPSCs from controls and BrS patients absent of pathogenic SCN5A mutations, even after challenge with ajmaline, highlighting future challenges in modelling the complex aetiology and pathophysiology of the disease.

The following are the supplementary data related to this article.

Movie 1 A spontaneously contracting cardiac cluster in a round-bottom 96 well plate generated from hiPSCs. The cluster shown is from iBR1-P5M-L9 at D41 of differentiation. Movie 1

Supplementary material Image 1

## Funding

This work was supported by a Barts Charity large project grant [Ref: 417/1615], University College London Hospital (UCLH) Biomedicine National Institute for Health Research (NIHR), The National Institute for Health Research Barts Cardiovascular Biomedical Research Unit, BHF programme grant (Ref:- RG/15/15/31742) and a Wellcome Trust grant to J.S.W. [WT 107469]. A.T. is supported by BHF grants SP/15/9/31605 and FS/12/59/29756. S.C.H is supported by a BHF Intermediate Basic Science Research Fellowship [FS/12/59/29756]. P.D.L. receives funding support from the UCLH Biomedicine NIHR.

## Conflicts of interest

None.
