# Supplementary material for: Ajmaline blocks INa and IKr without eliciting differences between Brugada syndrome patient and control human pluripotent stem cell-derived cardiac clusters
Source: Stem Cell Res. 2017 Dec;25:233–44. doi: 10.1016/j.scr.2017.11.003 (PMC5727153; doi:10.1016/j.scr.2017.11.003)
Supplement: Supplementary file 2 — Supplementary material [file mmc2.docx]

**Ajmaline blocks *I*_Na_ and *I*_Kr_ without eliciting differences between Brugada Syndrome patient and control human pluripotent stem cell-derived cardiac clusters**

**SUPPLEMENTARY FIGURES**

**Supplementary Figure S1. FP recordings and analysis of drug effects on iPS-HS1M cardiac clusters**

A) Screenshot of CardioMDA software, showing consistent signal over a 1 min recording following drug superfusion. B) FP recordings of iPS-HS1M cardiac clusters at baseline and after (black arrowhead) the highest dose of ajmaline (100 µM), mexiletine (100 µM) and dofetilide (10 µM). C) Flow cytometry analysis at day 21 from two parallel differentiation experiments showing an increase in TNNT2^+^ cells in clusters following seven days metabolic enrichment using sodium lactate. LE – lactate enrichment. D) Comparison of the effects of ajmaline on activation-recovery interval between standard and LE clusters. N= 8 electrodes measuring 5 (standard) or 4 (LE) clusters from 2 parallel differentiation experiments, ± s.e.m. Control readings of basal medium from Figure 2D have been overlaid. E) Mean beat rate of cardiac clusters, showing effect of increasing doses of ion channel inhibitors normalised to baseline ± s.e.m

**Supplementary Figure S2. Reprogramming of BrS patient hiPSCs**

A) RT-PCR analysis of pluripotency associated marker expression and absence of hStemCCa transgene expression in hPSC lines. B) Immunofluorescence staining in iBR1-P5M-L1 and control HUES7, with hPSC colonies on MEFs showing positive staining for pluripotency associated markers. Bar = 100 µm. C) G-band staining of iBR1-P5M-L1 at p15, exhibiting a normal karyotype. D) Immunofluorescence staining of hiPSC lines following 14 days of EB differentiation and outgrowth, showing expression of mesoderm (ACTA), neurectoderm (TUBB3), and endoderm (SOX17) markers. Bar = 100 µm.

**Supplementary Figure S3. Differentiation and analysis of hPSC cardiac clusters**

A) Day 14 count of spontaneously contracting clusters across hPSC lines. Error bars show ± s.e.m. where n≥ 2. *ND* – not done. B) Flow cytometry analysis of TNNT2 expression in hPSC cardiac clusters at day 21 of differentiation. Error bars show ± s.e.m. where n≥ 2. C) Baseline cFPD of different hPSC lines, based on the activation-recovery interval. Error bars show ± s.e.m., n= 8 to 11, see Supplementary Table S1. ** p≤ 0.01 vs P3M-N2 and P6M-L1, # p≤ 0.05 vs P3M-N2, one way ANOVA followed by Tukey HSD. D) Mean activation downslope gradient (minimum velocity, dV/dt_min_) of hPSC cardiac clusters following 100µM ajmaline, normalised to baseline. Error bars ± s.e.m., n= 8 to 11.

**
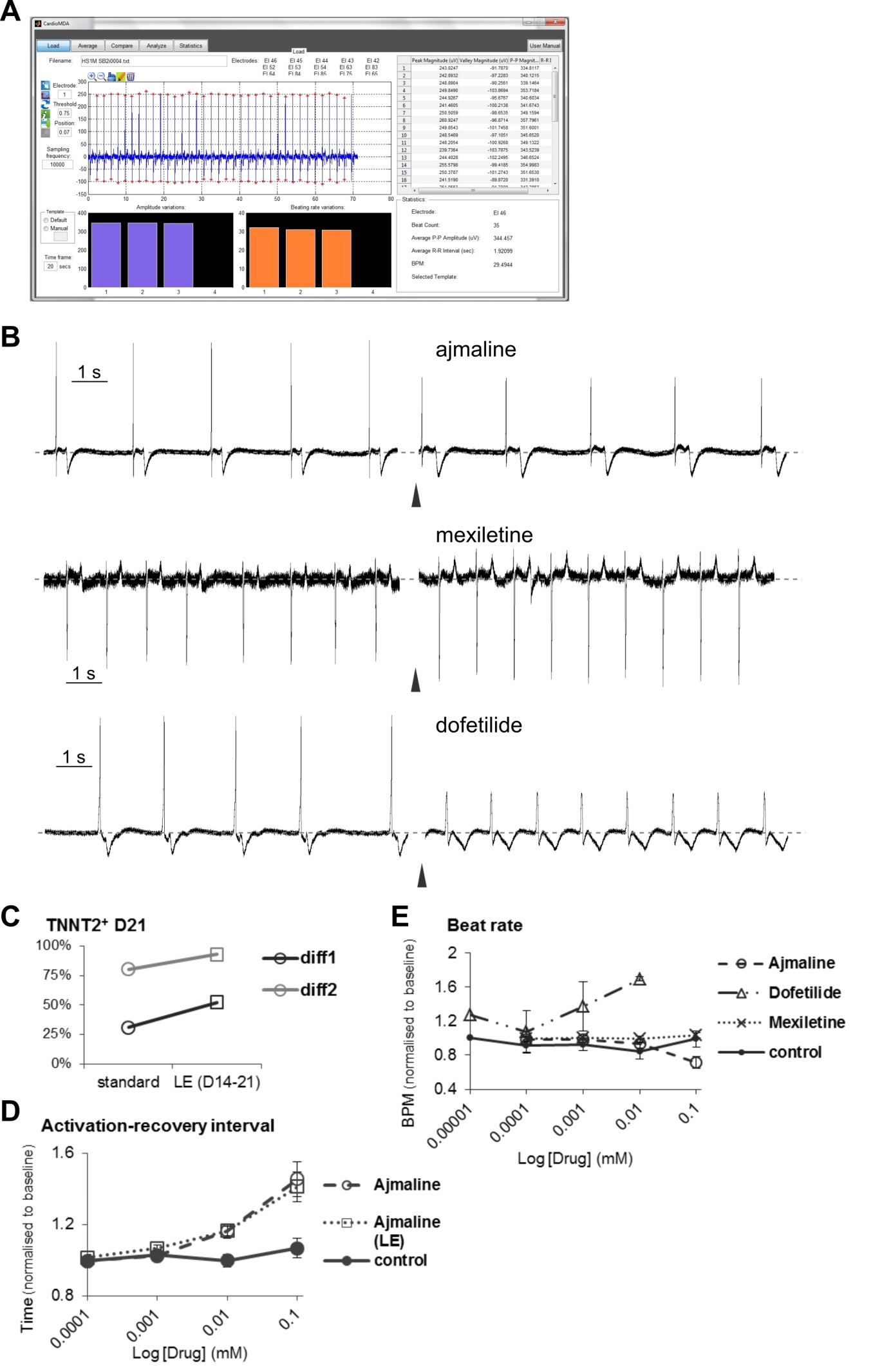
Supplementary Figure S1.**

**Supplementary Figure S2.
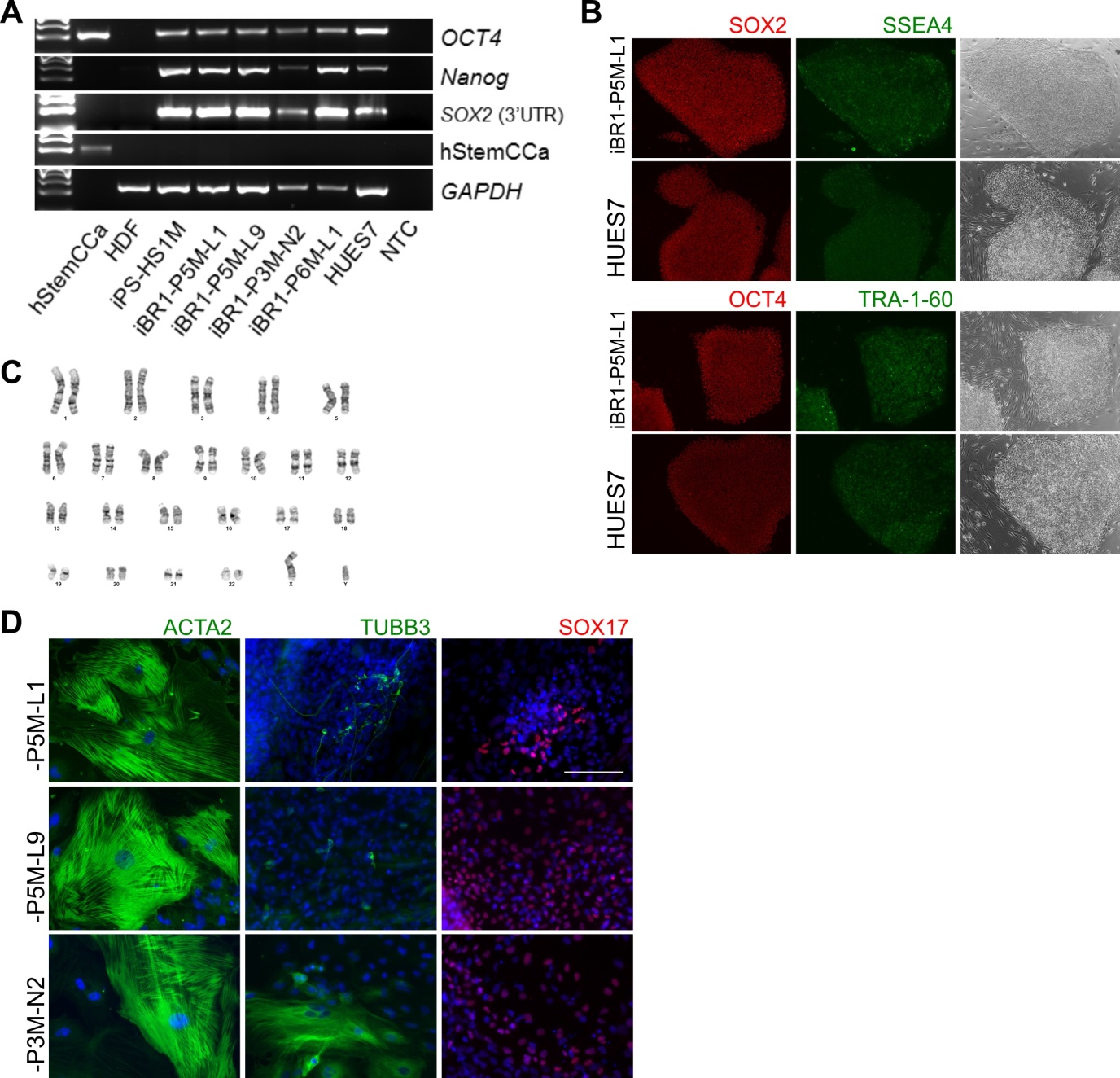
**

**Supplementary Figure S3.
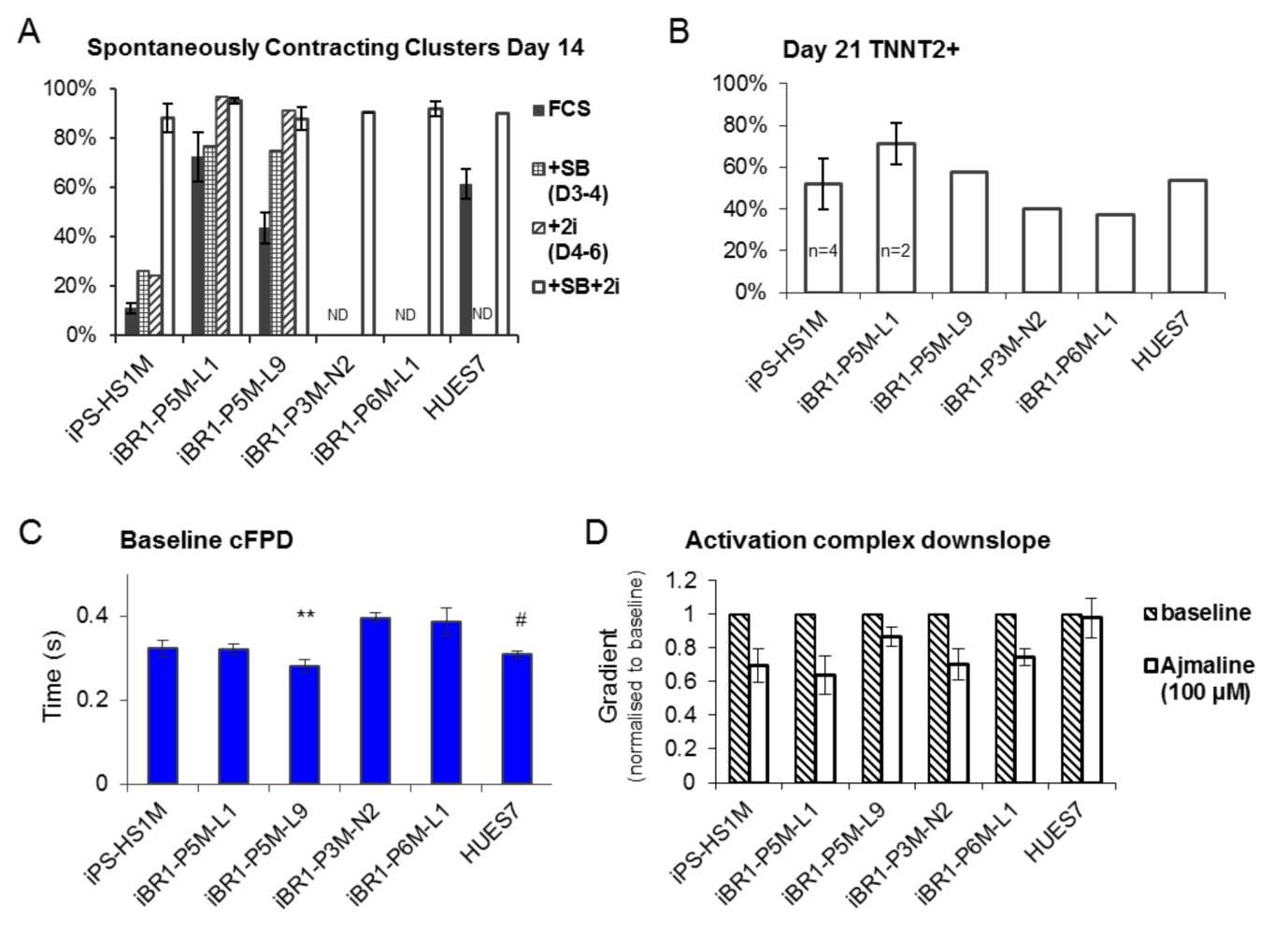
**

**SUPPLEMENTARY TABLES**

**Supplementary Table S1. Summary of MEA data from patient and control hPSC cardiac clusters during ajmaline treatment** (Below)

The number of differentiation experiments (diff.), clusters and electrodes used to generate mean cFPD is indicated.

|  | **Ajmaline dose** | **iPS-HS1M**  Diff. x3, clusters x7, electrodes x11 | **iBR1-P5M-L1**  Diff. x2, clusters x4, electrodes x9 | **iBR1-P5M-L9**  Diff. x1, clusters x5, electrodes x9 | **iBR1-P3M-N2**  Diff. x1, clusters x5, electrodes x8 | **iBR1-P6M-L1**  Diff. x1, clusters x6, electrodes x9 | **HUES7**  Diff. x2, clusters x5, electrodes x9 |
| --- | --- | --- | --- | --- | --- | --- | --- |
| **Mean cFPD**  (ms ±s.e.m.) | Baseline  100 nM  1 µM  10 µM  100 µM | 322.3 ±19.1  326.0 ±19.3  334.4 ±19.0  379.8 ±29.4  462.1 ±47.4 | 319.6 ±12.8  327.4 ±13.6  352.1 ±12.6  385.7 ±13.3  415.2 ±26.1 | 281.3 ±15.0  307.7 ±13.8  328.7 ±15.0  364.8 ±16.4  418.7 ±23.1 | 395.5 ±10.9  439.3 ±7.9  448.0 ±13.9  505.0 ±21.8  596.6 ±19.5 | 385.4 ±32.9  410.1 ±32.8  428.7 ±29.7  484.0 ±30.1  553.0 ±27.0 | 309.0 ±8.6  321.5 ±10.1  336.1 ±16.4  379.6 ±18.8  462.3 ±27.6 |
| **Relative mean cFPD**  (±s.e.m.) | Baseline  100 nM  1 µM  10 µM  100 µM | 1.000 ±0.059  1.011 ±0.060  1.037 ±0.059  1.178 ±0.091  1.434 ±0.147 | 1.000 ±0.040  1.024 ±0.043  1.102 ±0.040  1.207 ±0.042  1.299 ±0.082 | 1.000 ±0.053  1.094 ±0.049  1.169 ±0.053  1.297 ±0.058  1.488 ±0.082 | 1.000 ±0.028  1.111 ±0.020  1.133 ±0.035  1.277 ±0.055  1.508 ±0.049 | 1.000 ±0.085  1.064 ±0.085  1.112 ±0.077  1.256 ±0.078  1.435 ±0.070 | 1.000 ±0.028  1.041 ±0.033  1.088 ±0.053  1.229 ±0.061  1.496 ±0.089 |

**Supplementary Table S2. Formulation of hPSC cardiac differentiation media**

| **Component** | **Concentration** |  |  |
| --- | --- | --- | --- |
|  | **RGF** | **RS** | **RI** |
| RPMI (Sigma) | 1x | 1x | 1x |
| Poly-vinyl alcohol (Sigma) | 4 mg/ml | - | - |
| Pen/Strep (Gibco) | 1x | 1x | 1x |
| ITS (Gibco) | 1x | - | 1x |
| Chemically defined lipid (Gibco) | 1x | - | 1x |
| 1-Thioglycerol (Sigma) | 400 µM | 400 µM | 400 µM |
| BMP-4 (R&D) | 20 ng/ml | - | - |
| FGF2 (Peprotech) | 6 ng/ml | - | - |
| Y-27632 (Tocris) | 1 µM | - | - |
| Foetal calf serum (Gibco) | - | 20% | - |
| SB431542 (Tocris) | - | 5 µM (D3-4) | - |
| XAV939 (Sigma) | - | - | 10 µM (D4-6) |
| KY02111 (Tocris) | - | - | 10 µM (D4-6) |

**Supplementary Table S3. RT-PCR primer sequences**

Shown 5’ to 3’. * qRT-PCR was performed for 40 cycles at 60^o^C annealing temperature.

| Target | F | R | No. cycles, anneal temp * |
| --- | --- | --- | --- |
| GAPDH | GAAGGTGAAGGTCGGAGTC | GAAGATGGTGATGGGATTTC | 28, 60^O^C |
| Nanog | AGCCTCTACTCTTCCTACCACC | TCCAAAGCAGCCTCCAAGTC | 32, 56^O^C |
| OCT4 | GCGAACCAGTATCGAGAACC | GCCTCAAAATCCTCTCGTTG | 32, 60^O^C |
| SOX2 3’UTR | CATGTCCCAGCACTACCAGA | GGGTTTTCTCCATGCTGTTT | 32, 48^O^C |
| hStemCCa | GTACTCCTCGGTCCCTTTCC | GTGGAGAAAGATGGGAGCAG | 36, 56^O^C |
| GAPDH | ATGGGGAAGGTGAAGGTCG | TAAAAGCAGCCCTGGTGACC | * |
| GFRA2 | GCTTGGGGACCAGTGTCAT | CTTTGGAGTTGTTGGCCTTC | * |
| ISL1 | GACGGTGGCTTACAGGCTAA | GCGAAGTCGCTCAGTACTTTC | * |
| TNNT2 | TTCACCAAAGATCTGCTCCTCGCT | TTATTACTGGTGTGGAGTGGGTGTGG | * |
| MYH7 | TCGTGCCTGATGACAAACAGGAGT | ATACTCGGTCTCGGCAGTGACTTT | * |
| SCN5A | GGTCCTCACAGTCTTCTGCC | GCACTTGTGCCTTAGGTTGC | * |
| KCNH2 | GCCGTAAGTTCATCATCGCC | CAGAAGCCGTCGTTGCAGTA | * |

**SUPPLEMENTARY METHODS**

*Next generation sequencing (NGS) of patient and control genomic DNA*

Reads were aligned and variants identified according to best practice guidelines. Low quality (Q< 20, window_size 5) reads/bases were trimmed using prinseq-lite 0.20.4, and read quality was assessed using FastQC v0.10.1. High-quality reads were mapped to UCSC GRCh37/hg19 reference genome using Burrows-Wheeler Aligner (BWA) v0.7.10. Picard v1.117 and the Genome Analysis Toolkit (GATK) v3.2 were used to mark duplicate reads, to perform local realignment around indels, and to recalibrate base quality scores according to best practices. Alignment summary metrics and coverage and callability metrics were generated using Picard v1.117, SAMtools v0.2.0, Bedtools v2.11.1 and in-house Perl/Shell scripts. A base was considered ‘callable’ if sequenced with minimum read depth= 10×, base quality≥ 20 and mapping quality≥ 10. GATKv3.2 HaplotypeCaller and Unified Genotyper were used to call variants from reads mapped with quality≥ 8. Indels with QD<2.0, ReadPosRankSum < -20, FS > 200 were filtered out. SNPs with QD < 2.0, FS > 60.0, MQ < 40.0, MQRankSum < -12.5, ReadPosRankSum < -8.0 were filtered out. Variants were annotated using the Variant Effect Predictor.

*Single cell dissociation of hiPSC cardiac clusters*

Cardiac clusters were dissociated to monolayers between weeks 5 - 6 of differentiation. RI medium was removed, clusters washed in PBS and incubated in 0.25% trypsin/EDTA for 12-15 mins, gently pipetting after 9 and 12 mins. RI with 20% FCS was added to inactivate the trypsin, and cells were centrifuged for 5 mins at 500g and plated in RI + 10% FCS. Medium was replaced with RI (no FCS) after two days. To generate single cells, monolayers were washed twice with PBS and dissociated using TrypLE (Life Technologies) with incubation at 37^O^C for 8-10 minutes. The cells were pipetted gently six times to ensure dissociation and RI + 10% FBS was added. The dissociated cells were transferred to a falcon tube and centrifuged at 800 rpm for 3 mins to pellet the cells. The supernatant was removed and the cells resuspended in RI + 10% FCS. After resuspension the cells were seeded (at the desired density) onto glass coverslips that had been pre-coated with 0.1% gelatin. Twenty four hours later the cells were inspected for attachment and the media changed to RI. Cells were patch clamped between 3-9 days after seeding. Media was replaced every 3 days.

*RT-PCR*

Cell samples were collected and RNA extracted using Trizol (Thermo Fisher, USA), and reverse transcribed to complementary DNA (cDNA) using random hexamer primers and M-MLV reverse transcriptase (both from Promega, USA). Quantitative RT-PCR (qRT-PCR) was performed with SYBR green JumpStart (Sigma) using a Stratagene Mx3000P (Agilent, USA), with a 60^o^C annealing temperature and amplifying for 40 cycles followed by a dissociation curve. For primer sequences, see Supplementary Table S3. Qualitative or qRT-PCR product was confirmed by agarose gel electrophoresis.

*Immunofluorescence microscopy and flow cytometry*

Immunocytochemistry and flow cytometry analysis of hPSC-CMs at weeks 3-4 of differentiation was performed following dissociation or partial dissociation of clusters as described above. For immunofluorescence microscopy, cell cultures were fixed for 10 mins with 4% paraformaldehyde (Thermo fisher), followed by permeabilisation and blocking in phosphate buffered saline (PBS) containing 0.1% Triton X-100 (Sigma) and 2% bovine serum albumin (BSA) (Sigma). Primary and secondary antibodies were then added sequentially, followed by DAPI (Sigma). For Tra-1-60 live cell staining, fixation, blocking and permeabilisation steps were omitted, and primary and secondary antibodies added directly to the cell culture medium. Staining was visualised using an Axioplan or AxiVert 200M microscope with an Axio MRc camera (all from Zeiss, Germany). Images were processed using Image J. For flow cytometry, dissociated single cells were fixed for 10 mins with 4% paraformaldehyde, followed by permeabilisation for 10mins using cold 70% methanol. Cells were blocked using PBS with 1% BSA, then primary and secondary antibodies added sequentially. Cells were analysed using an LSRFortessa (BD Biosciences). Gating of positive staining was applied based on isotype IgG control antibody (Santa Cruz Biotechnology, USA) staining.

Antibodies used were anti- OCT4 goat IgG, Nanog goat IgG, SOX2 goat IgG, Tra-1-60 mouse IgM, SSEA4 mouse IgG (all from R&D, USA), TNNT2 mouse IgG (Abcam), and NKX2-5 rabbit IgG (Santa Cruz Biotechnology), with Alexa Fluor® secondary antibodies (Thermo Fisher).
